# Tuberculosis Notification Trends and Treatment Outcomes in Penitentiary and Civilian Health Care Sectors in the WHO European Region

**DOI:** 10.3390/ijerph18189566

**Published:** 2021-09-10

**Authors:** Andrei Dadu, Ana Ciobanu, Araksya Hovhannesyan, Natavan Alikhanova, Oleksandr Korotych, Elmira Gurbanova, Rafael Mehdiyev, Svetlana Doltu, Ogtay Gozalov, Sevim Ahmedov, Masoud Dara

**Affiliations:** 1 Regional Office for Europe, World Health Organization, 2100 Copenhagen, Denmark; araxhovhannesyan@gmail.com (A.H.); korotycho@who.int (O.K.); gozalovo@who.int (O.G.); daram@who.int (M.D.); 2 Health Primary Care Department, University of Medicine and Pharmacy ‘N. Testemitanu’, 2004 Chisinau, Moldova; anna.ciobanu@gmail.com; 3 Main Medical Department of the Ministry of Justice of Azerbaijan Republic, Baku AZ1000, Azerbaijan; natavan_x@yahoo.com (N.A.); medical.department@justice.gov.az (R.M.); 4 WHO Collaborating Centre on Prevention and Control of TB in Prisons, Baku AZ1000, Azerbaijan; elmiragurbanova@gmail.com; 5 Council for Preventing and Eliminating Discrimination and Ensuring Equality, 2004 Chisinau, Moldova; svetlana.doltu@gmail.com; 6 United States Agency for International Development, Arlington, VA 22202, USA; sahmedov@usaid.gov

**Keywords:** tuberculosis, prisons, notification, outcomes, WHO European Region

## Abstract

Setting: Tuberculosis (TB) morbidity in penitentiary sectors is one of the major barriers to ending TB in the World Health Organization (WHO) European Region. Objectives and design: a comparative analysis of TB notification rates during 2014–2018 and of treatment outcomes in the civilian and penitentiary sectors in the WHO European Region, with an assessment of risks of developing TB among people experience incarceration. Results: in the WHO European Region, incident TB rates in inmates were 4–24 times higher than in the civilian population. In 12 eastern Europe and central Asia (EECA) countries, inmates compared to civilians had higher relative risks of developing TB (RR = 25) than in the rest of the region (RR = 11), with the highest rates reported in inmates in Azerbaijan, Kazakhstan, Kyrgyzstan, Republic of Moldova, Russian Federation, and Ukraine. The average annual change in TB notification rates between 2014 and 2018 was −7.0% in the civilian sector and −10.9% in the penitentiary sector. A total of 15 countries achieved treatment success rates of over 85% for new penitentiary sector TB patients, the target for the WHO European Region. In 10 countries, there were no significant differences in treatment outcomes between civilian and penitentiary sectors. Conclusion: 42 out of 53 (79%) WHO European Region countries reported TB data for the selected time periods. Most countries in the region achieved a substantial decline in TB burden in prisons, which indicates the effectiveness of recent interventions in correctional institutions. Nevertheless, people who experience incarceration remain an at-risk population for acquiring infection, developing active disease and unfavourable treatment outcomes. Therefore, TB prevention and care practices in inmates need to be improved.

## 1. Introduction

In 2019 in the world, 10 million people developed TB disease, and 1.4 million died from TB. Although WHO European Region carries only 3% of the global burden of tuberculosis (TB), it has one of the highest proportions of multidrug-resistant TB (MDR-TB). In 2019, an estimated 246,000 incident TB cases occurred in countries of the WHO European Region, equivalent to an average incidence of 26 cases per 100,000 population [1].

Over the last century, global control efforts have reduced the worldwide burden of tuberculosis (TB) [1]. Nevertheless, TB morbidity in the penitentiary sector (The term “penitentiary sector” includes jails, remand/detention/pre-trial centres and prisons) remain a significant barrier to ending TB in the World Health Organization (WHO) European Region by 2030 [1,2]. Worldwide, more than 10 million people are inmates, with around half located in Brazil, China, the Russian Federation and the United States of America [3,4]. According to the United Nations’ estimates of national population levels, the known prison population of the world increased by 3.7% between 2015 and 2018. In the WHO European Region, the most substantial increases in prison populations were observed in Turkey (an increase of 31%), Belarus (19%) and Italy (14%). However, prison populations decreased in Romania (a decrease of 22%), Ukraine (19%) and the Russian Federation (10%) during the same period [5]. 

TB prevention and care services in prisons are described as often being inadequate and poorly integrated with civilian services, and prison inmates consistently have higher risks of developing active TB and dying from TB than the general population, owing to poor conditions in prisons, such as overcrowding, inadequate ventilation, malnutrition, poor hygiene and poor health care [6,7,8,9,10,11,12]. Additionally, inmates often come from communities, which are at an increased risk of TB or HIV infections [2,8,13].

The objectives of this study were to describe the diversity of notification of incident TB cases (notification rate of incident TB cases—number of new and relapse tuberculosis cases registered and reported per 100,000 population [1]) and their trends in the civilian and penitentiary sectors between 2014 and 2018; the treatment outcomes in the penitentiary versus the civilian sectors, and to estimate the relative risks of developing active TB for prison inmates (inmates—includes people experience incarceration, detainees and convicts) in comparison to civilian population in the WHO European Region.

## 2. Methods and Materials

This is a retrospective descriptive study analysing magnitude and time-series trends in the notification of new and relapse TB cases and TB treatment outcomes in the civilian and penitentiary sectors in the WHO European Region, based on data reported by WHO European Region Member States to The WHO global TB data collection system [14] between 2012 and 2018, inclusively.

### 2.1. Setting

The WHO European Region consists of 53 Member States covering a vast geographical region from the Atlantic to the Pacific oceans and from the Mediterranean to the Baltic Sea [15]. Our study focused on comparative analyses of TB indicators in eastern Europe and central Asia (EECA) countries as well as of those from the rest of the region. The EECA region is made up of 12 of the 15 countries, which were formerly part of the Soviet Union, excluding the Baltics, and which are located in the east of the WHO European Region.

### 2.2. Study Population and Design

Data were collected for new and relapse TB cases and their outcomes from the civilian and penitentiary sectors reported in WHO European Region countries. Three selection criteria were applied: (1) countries that provided at least one report on new and relapse TB cases in both the civilian and penitentiary sectors between 2014 and 2018; (2) countries that provided at least two data points on new and relapse TB cases in both civilian and penitentiary sectors between 2014 and 2019 for enabling analysis of the trend; (3) countries that reported outcomes for TB cases on first-line drug (FLD) treatment schemes in both the civilian and penitentiary sectors for at least one cohort between 2012 and 2016.

### 2.3. Data Variables and Sources

Data were obtained from 3 sources: (1) The WHO global TB data collection system [14] has an extended set of indicators for TB in European Region prisons, and data were extracted on: prison populations, the numbers of new and relapse TB cases in the civilian and penitentiary sectors for 2014 to 2018, and treatment outcomes for patients on FLD treatment schemes in the civilian and penitentiary sectors for 2012 to 2016; (2) total population estimates were extracted from World Population Prospects [16]; and (3) prison population estimates were taken from the World Prison Brief [17] for countries whose prison population data were missing from The WHO global TB data collection system.

### 2.4. Analysis and Statistics

For each country, we calculated annual notification rates per 100,000 population of new and relapse tuberculosis cases in civilian and penitentiary sectors separately. The Average Annual Percent Change (AAPC) was calculated by fitting a least-squares regression line to the natural logarithm of the rates, using the calendar year as a regressor variable. 

As a measure of the effect of exposure to a prison setting on the risk of development of TB we computed the Relative Risk of TB in prison in reference to the civilian population and the corresponding confidence interval. Results were considered significant if the confidence interval did not include 1. The statistical analysis was performed using the online calculator VassarStats [18]. TB patients who were successfully treated or completed TB treatment were considered to have a favourable outcome; those who failed to complete treatment, were lost to follow-up or who died during the TB treatment were considered to have an unfavourable outcome, as per WHO standard definitions [19]. TB cases with no reported treatment outcomes were excluded from the analysis. We analysed the notification rate of incident TB cases and TB treatment outcomes (unfavourable versus favourable) for the civilian and penitentiary sectors.

## 3. Results

### 3.1. Notification Rate of Incident TB Cases and Relative Risks of Developing TB Disease in the Penitentiary Sector Compared with the Civilian Sector

**Figure 1:**
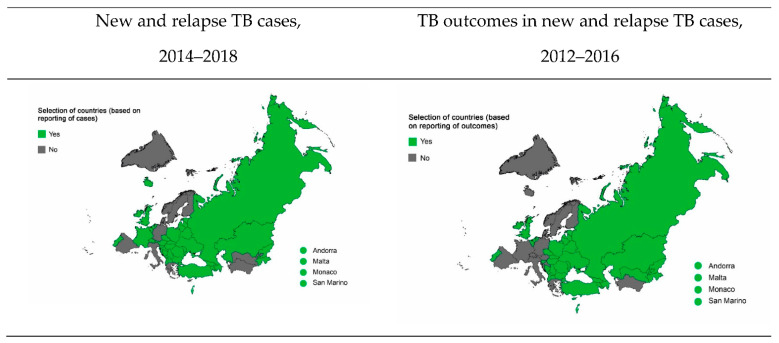
Countries/states selected for case analysis based on the availability of TB reporting forms. TB: tuberculosis; WHO: World Health Organization.

Out of the 53 countries of the WHO European Region, 42, including 10 from the EECA region, reported the number of new and relapse TB cases in the civilian and penitentiary sectors at least once in the five-year period between 2014 and 2018. During this time, 11 (21%) countries did not provide any reports on TB in prisons (Table 1 and Figure 1).

**Figure 2:**
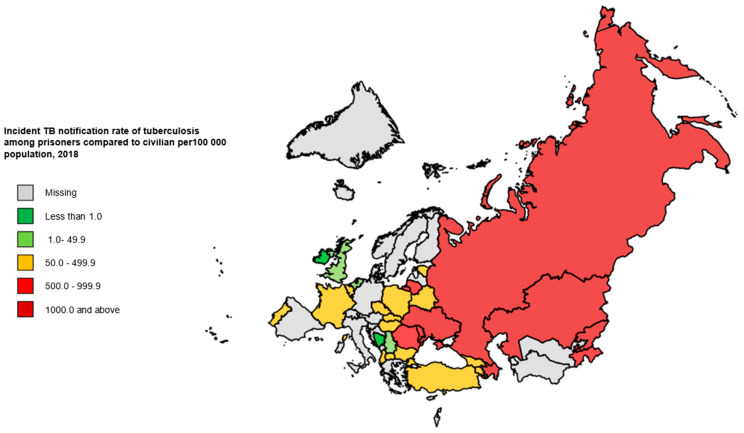
Notification rate of incident TB cases in the penitentiary sector per 100,000 population, WHO European Region, 2018. TB: tuberculosis; WHO: World Health Organization.

Table 2 shows the notification rate of incident TB cases (The notification rate of incident TB cases is the number of new and relapse tuberculosis cases reported per 100,000 population [1]) and percentage annual changes in notification rate of incident TB cases in the civilian and penitentiary sectors during 2014–2018 for the countries included in this study. In the penitentiary sectors of seven countries, all of which are in the EECA region, incident TB rates of more than 1000 per 100,000 population were reported: Azerbaijan, Kazakhstan, Kyrgyzstan, the Republic of Moldova, the Russian Federation, Tajikistan and Ukraine (Table 2 and Figure 2).

**Figure 3:**
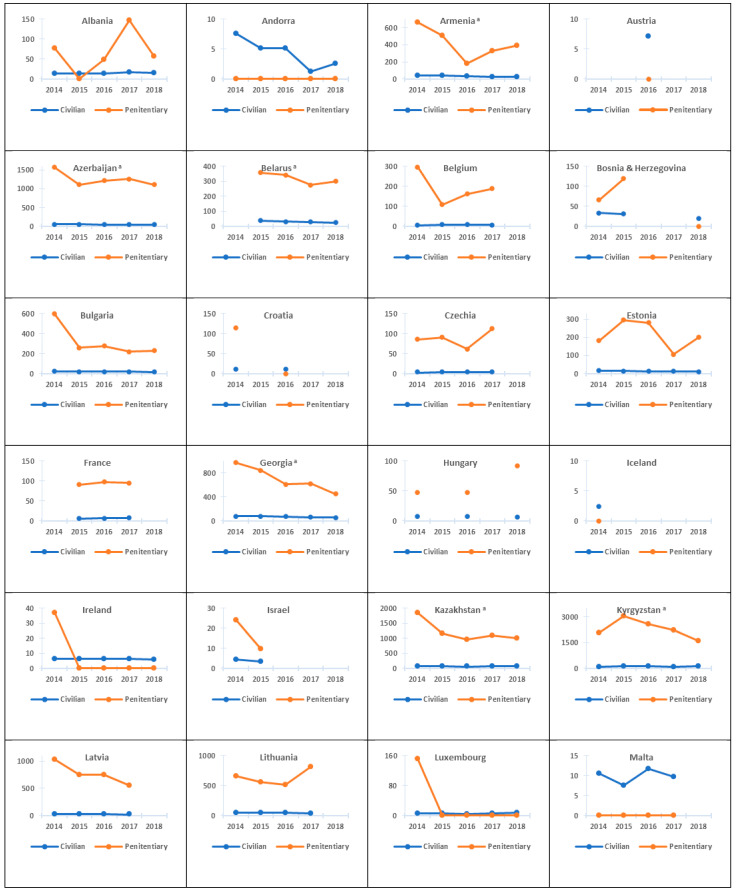

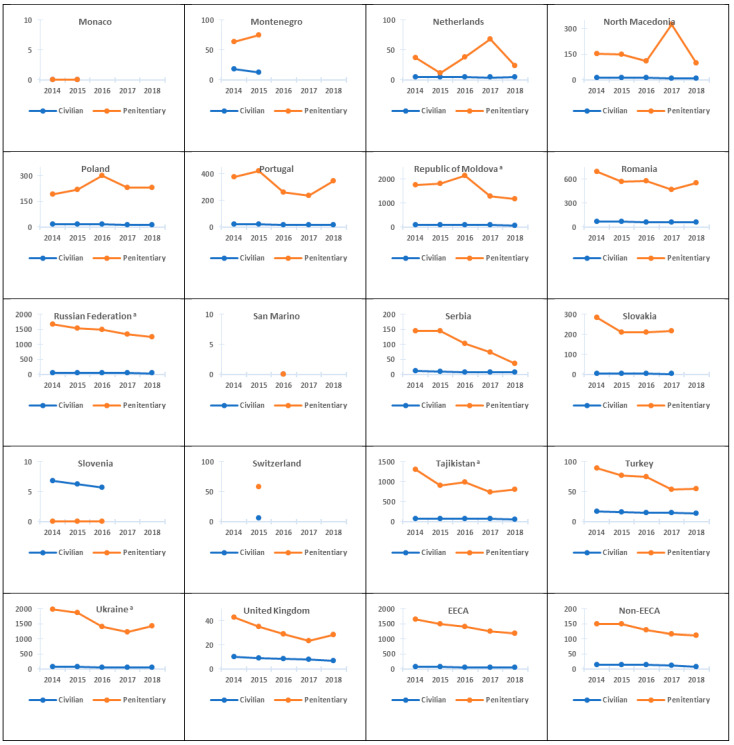
Trends in TB notification rates in the civilian and penitentiary sectors, WHO European Region, 2014–2018. TB: tuberculosis; WHO: World Health Organization. ^a^ EECA country. Note: TB notification rate: number of new and relapse tuberculosis cases registered and reported per 100,000 population.

There was observed a decreasing trend in the notification of new TB cases and relapses both in the penitentiary and in the civil sector in 2014–2018 (Table 2 and Figure 3).

In the 42 countries analysed, the average annual change in incident TB rates during the study period was −7.0% in the civilian sector and −10.9% in the penitentiary sector. The decline in incident TB rates among inmates in the nine EECA countries included in this study should be noted (from −6.0% in Kyrgyzstan to −16.5% in Georgia) (Table 2).

TB cases registered in prison’s inmates accounted for approximately 7% for all notified new and relapse TB patients in EECA countries, with the highest level in the Russian Federation (10%); in comparison, in the other countries in the region the proportion was 1.5%, with the highest level in Slovakia (6.3%) in 2014–2018. 

**Figure 4:**
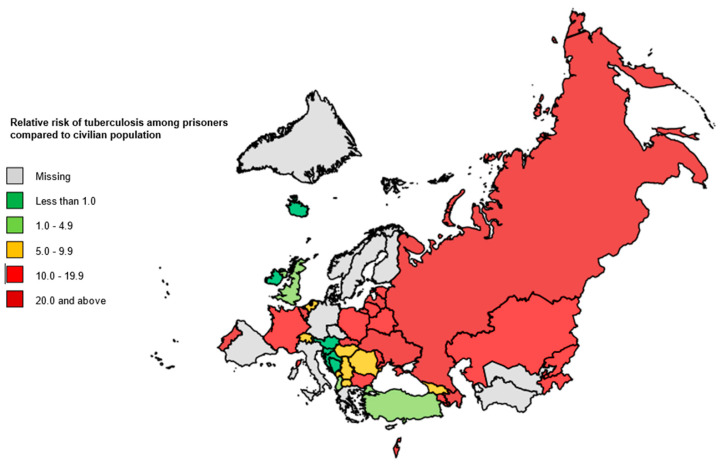
Relative risks of developing TB among prisons’ inmates compared to civilian population, WHO European Region, 2014–2018. TB: tuberculosis; WHO: World Health Organization.

Prison’s inmates in the Russian Federation and Slovakia had the highest risks of developing an active TB disease compared with their respective civilian populations (RR = 25, confidence interval CI: 25–26, and RR = 57 (CI: 35–92)), respectively, in the last reported year. (Table 3 and Figure 4).

### 3.2. Treatment Outcomes in TB Patients on First-Line Drug (FLD) Treatment Schemes

A total of 39 (74%) countries in the WHO European Region reported treatment outcomes for at least one cohort of TB patients, both civilians and inmates, who started on one of the FLD treatment schemes between 2012 and 2016 (Table 1 and Figure 1). Table 4 shows both the favourable and unfavourable treatment outcomes for civilians and inmates in these 39 countries.

Our study highlights a few countries where there were higher levels of unfavourable outcomes for inmates when compared with civilians, for example, Cyprus (100% vs. 0%), the Netherlands (29% vs. 9%) and Kazakhstan (21% vs. 9%). On the other hand, a higher proportion of unfavourable treatment outcomes among civilians than among inmates had been registered in the Czech Republic (28% vs. 8%), Andorra (25% vs. 0%), Estonia (20% vs. 0%), Armenia (19% vs. 0%), and Slovenia (16% vs. 0%).

A total of 12 of the 39 countries achieved TB treatment success rates of over 85% among inmates. In two EECA countries, Belarus and Tajikistan and in five other countries, Bulgaria, Latvia, Montenegro, Romania, Slovakia, the favourable outcomes were more than 85% in both sectors civilian and penitentiary.

## 4. Discussion

Recent systematic review by Cords et al. revealed a concerning scale of TB burden among people experiencing incarceration in different parts of the world and highlighted the high risk of contracting M tuberculosis infection and developing active disease, compared to the general population [12].

This is the first standardized study on TB morbidity and its treatment outcomes monitoring in the penitentiary sectors of such a scale in the WHO European Region. The main finding of our study is that from 2014 to 2018 the annual incident TB notification rates in prisons across the European Region decreased much faster than in the civilian population, which most likely reflects the decline of true burden in the prison populations. This finding highlights the positive impacts of the TB control interventions carried out by national governments, with the support of international agencies [20,21]. Another finding of our study is that, even though the annual decline of the TB burden in WHO European Region prisons was faster than in the civilian sector, the risk of developing TB disease in prisons is up to 57 times higher compared to the civilian sector. The increased risk of TB for inmates in EECA countries is a known feature of the region and has been previously described in several studies [11,22,23,24]. High prevalence of active TB disease in correctional facilities is fuelled by intra-institutional transmission due to prolonged stays in overcrowded facilities with poor ventilation, along with risk factors, which amplify the risk of TB disease, such as HIV, malnutrition, diabetes, smoking, a history of alcohol and illicit drug consumption, and former TB disease [9,10,25,26]. Theoretically, prison settings offer great opportunities for TB control, and there are practical examples from the region’s prisons in which significant improvements in their TB and rifampicin-resistant-TB burdens have been reported, and WHO-recommended screening, diagnostics, treatment, and linkage to civilian health care is ensured [27,28,29,30]. The high TB morbidity rates in the region’s prisons, of up to 1623 per 100,000 population in 2018, underline the need for substantial improvements in TB control among inmates through wider application of the best practices in the field.

In the majority of EECA countries, treatment success rates for TB in inmates were lower compared with rates in civilian populations, which was not evident for the other countries in the region. This emphasizes the critical need for improvements in the TB services available to inmates. Although the specific reasons for unfavourable treatment outcomes in inmates were not analysed in this study, there is evidence that high drug-resistance rates, insufficient laboratory diagnosis capacities and weak integrations between civilian and prison healthcare services, including ensuring the continuity of TB treatment after release from prison, are major factors leading to poor treatment outcomes in prisons [6,31].

Decarceration and other countries’ justice reforms that lead to it would reduce overcrowding, which is a major environmental factor for tuberculosis transmission, and would significantly reduce TB burden and its rising rates in prisons. Meanwhile, improving the TB situation and treatment outcomes for inmates can only be achieved with governmental commitment, inter-department cooperation for ensuring interventions equivalent to those in the civilian system and in close collaboration with it, and partnerships with civil society organizations. National tuberculosis programmes (NTPs) should develop operational plans and policies that optimize TB control in prisons and for inmates after their release and strengthen the capacities of prison health units for TB case management. Improving treatment outcomes for inmates will also prevent transmission of disease to other inmates, prison staff and community members. The End TB Strategy goals [32] will not be met without the implementation of effective measures in prisons where there are a large number of people who are vulnerable to TB and who engage in behaviours, which also put them at high risk for HIV infection.

One of the limitations of our current study is immediately apparent from the observation of the poor reporting of TB in prisons: in the 5-year study period, there was no available data for 11 WHO European Region countries. In addition, huge fluctuations in the reported annual incident TB rates in prisons reflect uncontrolled epidemics. It is important to note that poor TB reporting affects TB morbidity statistics and, consequently, TB estimates at national and international levels.

This study revealed that some high TB burden countries, such as Turkmenistan and Uzbekistan, have not reported any TB cases among inmates. Cooperation between the institutions responsible for health care in penitentiary systems and the ministries of health should further improve to allow proper TB recording and reporting in both the civilian and penitentiary systems of all countries in the WHO European Region.

## 5. Conclusions

This review provides an overview of active TB in prisons in the WHO European Region. The completeness of TB reporting for prisons by NTPs was 79% (42 out of 53 countries from the WHO European Region). Our analysis highlights the vulnerability of inmates to TB and emphasises the necessity of improving TB prevention and care policies and their practical application in prisons with respect to active TB detection, infection control, TB treatment and continuity of care. Most countries achieved a substantial decline of TB burden in prisons, which indicates the effectiveness of recent interventions in correctional institutions. These results provide the basis for an understanding that TB prevention and care in prisons should be elevated to be a health care priority and should facilitate intersectional collaboration between civilian health authorities and prison administrations to enable ending TB in the WHO European Region.

### 5.1. Supporting Information Captions

#### 5.1.1. Evidence Available Prior to This Study

TB surveillance data from the WHO European Region were collected annually from countries via The WHO global TB data collection system [14]. The WHO Regional Office for Europe and the European Centre for Disease Prevention and Control (ECDC) have jointly coordinated the collection and analysis of TB surveillance data in the WHO European Region, aiming to ensure data timeline consistency and comparability, pan-European coverage and avoidance of data duplication [1].

#### 5.1.2. Added Value of the Study

This article provides a full cascade analysis of the TB burden and treatment outcomes at the regional level, designated by countries and subregional groupings.

#### 5.1.3. Implications of All Available Evidence

Further efforts should be made to reduce TB infection transmission, the development of active TB and acquisition of data on treatment outcomes either via WHO data collection or reports from individual countries. In particular, more attention needs to be placed on addressing the known risk factors associated with TB.

## Figures and Tables

**Table 1:** Completeness of TB reporting for civilian and penitentiary sectors via The WHO global TB data collection system, WHO European Region countries.

**a. Number of New and Relapse TB Cases, 2014–2018**											
**Country**	**Civilian Sector**					**Penitentiary Sector**					**Status**
	**2014**	**2015**	**2016**	**2017**	**2018**	**2014**	**2015**	**2016**	**2017**	**2018**	
Albania	404	415	412	495	437	4	0	3	8	3	Y
Andorra	6	4	4	1	2	0	0	0	0	0	Y
Armenia ^a^	1303	1151	1018	825	720	26	20	9	16	14	Y
Austria	−	−	619	−	−	−	−	0	−	−	Y
Azerbaijan ^a^	5490	5228	4905	4975	4822	298	228	254	256	216	Y
Belarus ^a^	−	3658	3090	2684	2253	−	107	121	97	106	Y
Belgium	851	916	967	896	−	35	12	19	20	−	Y
Bosnia and Herzegovina	1194	1091	−	−	666	2	4	−	−	0	Y
Bulgaria	1774	1599	1503	1392	1307	51	20	22	16	16	Y
Croatia	491	−	449	−	−	5	−	0	−	−	Y
Cyprus	−	−	−	−	−	−	−	−	−	−	N
Czechia	458	489	497	474	−	16	19	14	25	22	Y
Denmark	−	−	−	−	−	−	−	−	−	−	N
Estonia	230	197	180	168	140	6	9	8	3	5	Y
Finland	−	−	−	−	−	−	−	−	−	−	N
France	−	4433	4610	4774	−	−	61	65	65	−	Y
Georgia ^a^	3099	3070	2926	2539	2272	101	82	57	58	43	Y
Germany	−	−	−	−	−	−	−	−	−	−	N
Greece	−	−	−	−	−	−	−	−	−	−	N
Greenland	−	−	−	−	−	−	−	−	−	−	N
Hungary	789	−	728	−	−	10	−	9	−	−	Y
Iceland	8	−	−	−	−	0	−	−	−	−	Y
Ireland	292	295	293	301	294	5	0	0	0	0	Y
Israel	356	278	−	−	−	5	2	−	−	−	Y
Italy	−	−	−	−	−	−	−	−	−	−	N
Kazakhstan ^a^	14,282	13,423	11,838	12,063	12,479	962	583	484	386	353	Y
Kyrgyzstan ^a^	6233	6779	6810	6488	6198	157	248	216	199	140	Y
Latvia	685	664	609	522	−	53	33	32	21	−	Y
Lithuania	1424	1354	1312	1214	−	57	41	35	54	−	Y
Luxembourg	37	30	29	32	42	1	0	0	0	0	Y
Malta	45	32	50	42	−	0	0	0	0	−	Y
Monaco	0	0	−	−	−	0	0	−	−	−	Y
Montenegro	112	79	−	−	−	1	1	−	−	−	Y
Netherlands	798	845	863	757	784	16	5	14	19	7	Y
North Macedonia	280	278	260	206	214	4	4	3	10	3	Y
Norway	−	−	−	−	−	−	−	−	−	−	N
Poland	6387	6065	5927	5365	5025	152	172	216	170	171	Y
Portugal	2198	2053	1833	1728	1812	53	61	39	32	44	Y
Republic of Moldova ^a^	3937	3484	3398	3259	2933	121	124	173	99	89	Y
Romania	14,652	14,064	12,633	12,205	11,472	209	161	157	105	114	Y
Russian Federation ^a^	91,025	89,218	82,797	76,344	70,967	11,315	10,372	9610	8166	7291	Y
San Marino	−	−	0	0	0	−	−	0	0	0	Y
Serbia	969	864	744	730	637	15	15	11	8	4	Y
Slovakia	299	291	264	210	−	21	17	17	18	−	Y
Slovenia	142	129	118	−	−	0	0	0	−	−	Y
Spain	−	−	−	−	−	−	−	−	−	−	N
Sweden	−	−	−	−	−	−	−	−	−	−	N
Switzerland	−	527	−	−	−	−	−	4	−	−	Y
Tajikistan ^a^	5677	5804	5866	5794	5605	130	90	99	101	121	Y
Turkey	12,966	12,413	12,035	11,696	11,421	142	137	151	125	155	Y
Turkmenistan ^a^	−	−	−	−	−	−	−	−	−	−	N
Ukraine ^a^	30,245	28,974	28,133	26,485	25,745	1456	1177	919	744	767	Y
United Kingdom	6581	5821	5766	5226	−	43	33	27	22	−	Y
Uzbekistan ^a^	−	−	−	−	−	−	−	−	−	−	N
**b. Number of TB Patients Who Started on One of the FLD Treatment Schemes, 2012–2016**											
**Country**	**Civilian Sector**					**Penitentiary Sector**					**Status**
	**2012**	**2013**	**2014**	**2015**	**2016**	**2012**	**2013**	**2014**	**2015**	**2016**	
Albania	405	470	402	409	406	2	2	4	0	3	Y
Andorra	9	0	6	4	4	0	5	0	0	0	Y
Armenia ^a^	1341	1228	1202	896	861	9	23	26	14	8	Y
Austria	−	−	−	−	−	−	−	−	−	−	N
Azerbaijan ^a^	4341	3973	1389	1292	1270	275	321	234	183	194	Y
Belarus ^a^	3298	2935	2648	2458	2076	127	99	58	67	47	Y
Belgium	855	856	833	895	955	30	22	34	10	18	Y
Bosnia and Herzegovina	−	1257	1194	1091	907	−	4	2	1	0	Y
Bulgaria	2136	1878	1744	1578	1488	44	25	45	20	22	Y
Croatia	−	−	−	−	−	−	−	−	−	−	N
Cyprus	−	−	−	−	55	−	−	−	−	1	Y
Czechia	536	452	453	481	492	20	16	14	18	13	Y
Denmark	−	−	−	−	−	−	−	−	−	−	N
Estonia	218	210	189	163	158	4	16	5	9	8	Y
Finland	−	−	−	−	−	−	−	−	−	−	N
France	−	−	−	−	−	−	−	−	−	−	N
Georgia ^a^	3245	2994	2781	2780	2666	393	104	81	61	49	Y
Germany	−	−	−	−	−	−	−	−	−	−	N
Greece	−	−	−	−	−	−	−	−	−	−	N
Greenland	−	−	−	−	−	−	−	−	−	−	N
Hungary	1161	1016	−	847	−	11	14	−	4	−	Y
Iceland	−	−	−	−	−	−	−	−	−	−	N
Ireland	336	345	283	265	286	2	1	5	0	0	Y
Israel	506	295	317	261	−	3	10	5	2	−	Y
Italy	−	−	−	−	−	−	−	−	−	−	N
Kazakhstan ^a^	15,514	13,400	11,791	13,372	−	761	1056	682	634	−	Y
Kyrgyzstan ^a^	−	5533	5610	5969	5910	−	125	121	170	162	Y
Latvia	823	764	631	609	560	49	40	44	33	32	Y
Lithuania	1428	1347	1238	1183	1126	31	45	44	36	26	Y
Luxembourg	−	−	37	−	−	−		1	−	−	Y
Malta	41	49	0	0	0	0	0	0	0	0	Y
Monaco	−	3	0	−	−	−	0	0	−	−	Y
Montenegro	107	119	112	79	−	0	0	1	1	−	Y
Netherlands	906	796	782	833	850	18	20	14	4	14	Y
North Macedonia	345	309	277	278	260	1	8	4	4	3	Y
Norway	−	−	−	−	−	−	−	−	−	−	N
Poland	7057	6791	6369	6054	5904	204	220	131	142	195	Y
Portugal	2493	2336	2198	2053	1833	46	−	−	61	39	Y
Republic of Moldova ^a^	4073	3747	3358	2903	2909	130	142	101	89	139	Y
Romania	16,313	15,048	14,321	13,747	12,304	112	140	204	161	155	Y
Russian Federation ^a^	80,594	71,674	67,146	71,317	64,591	9072	11,627	9990	9107	8546	Y
San Marino	−	−	−	0	−	−	−	−	0	−	Y
Serbia	1171	1163	1032	868	722	26	21	13	14	11	Y
Slovakia	323	368	298	288	262	20	27	20	17	17	Y
Slovenia	138	139	142	129	−	0	0	0	0	−	Y
Spain	−	−	−	−	−	−	−	−	−	−	N
Sweden	−	−	−	−	−	−	−	−	−	−	N
Switzerland	−	−	−	−	−	−	−	−	−	−	N
Tajikistan ^a^	5664	5151	5047	5222	5254	147	112	102	76	70	Y
Turkey	13,409	13,047	12,791	12,219	11,851	126	123	142	143	166	Y
Turkmenistan ^a^	−	−	−	−	−	−	−	−	−	−	N
Ukraine ^a^	29,346	28,016	21,270	23,015	21,618	1582	1710	1024	877	997	Y
United Kingdom	8106	7260	6469	5773	5649	35	33	43	29	26	Y
Uzbekistan ^a^	13,783	−	−	−	−	349	−	−	−	−	Y

−: not reported; EECA: east European central Asia; N: excluded from the analysis; TB: tuberculosis; Y: included in the analysis; WHO: World Health Organization ^a^ EECA country.

**Table 2:** Notification rate of incident TB cases and year-to-year percentage changes in TB notification rates, WHO European Region, 2014–2018.

**a. Civilian Sector**										
**Country**	**Notification Rate of Incident TB Cases per 100,000 Population**					**Annual Change in Notification Rate of Incident TB Cases (%)**				
	**2014**	**2015**	**2016**	**2017**	**2018**	**2014–2015**	**2015–2016**	**2016–2017**	**2017–2018**	**Average**
Albania	13.9	14.2	14.1	16.9	15.2	2.6	−0.8	18.2	−10.8	2.3
Andorra	7.6	5.1	5.2	1.3	2.6	−39.0	0.9	−138.2	69.3	−23.5
Armenia ^a^	44.9	39.5	34.9	28.2	24.4	−12.8	−12.5	−21.2	−14.4	−14.1
Azerbaijan ^a^	57.9	54.5	50.5	50.7	48.6	−6.1	−7.5	0.4	−4.4	−4.3
Belarus ^a^		38.7	32.7	28.5	23.9		−16.8	−14.0	−17.3	−14.8
Belgium	7.6	8.1	8.5	7.8	7.8	6.7	4.8	−8.3	−0.5	0.7
Bosnia and Herzegovina	33.5	30.8			20.1	−8.4				−12.0
Bulgaria	24.6	22.3	21.1	19.7	18.1	−9.8	−5.5	−7.0	−8.4	−7.4
Croatia	11.5		10.7							−3.9
Czech Republic	4.3	4.6	4.7	4.5	3.9	6.5	1.6	−4.8	−14.2	−2.7
Estonia	17.5	15.0	13.7	12.9	10.6	−15.3	−8.8	−6.7	−19.3	−11.8
France		6.9	7.1	7.4	7.3		3.5	3.1	−1.3	1.8
Georgia ^a^	77.8	77.9	74.7	65.1	56.9	0.1	−4.1	−13.8	−13.4	−7.5
Hungary	8.1		7.5		6.1					−6.9
Ireland	6.2	6.3	6.2	6.3	6.1	0.7	−1.3	1.8	−3.6	−0.6
Israel	4.5	3.5				−26.3				−26.3
Kazakhstan ^a^	81.9	75.8	66.0	66.4	68.2	−7.7	−13.9	0.6	2.8	−4.5
Kyrgyzstan ^a^	108.1	115.7	114.5	107.5	120.8	6.8	−1.1	−6.3	11.7	2.8
Latvia	34.1	33.4	31.0	26.8		−2.0	−7.5	−14.4		−7.7
Lithuania	48.2	46.3	45.2	42.1	36.4	−4.1	−2.4	−7.2	−14.6	−6.8
Luxembourg	6.7	5.3	5.0	5.5	7.0	−22.8	−5.0	8.5	23.7	1.1
Malta	10.6	7.5	11.7	9.8	12.5	−34.6	44.2	−17.8	25.0	4.3
Monaco	0.0	0.0				0.0				0.0
Montenegro	17.9	12.6				−35.0				−35.0
Netherlands	4.7	5.0	5.1	4.5	4.6	5.4	1.8	−13.4	3.4	−0.7
North Macedonia	13.5	13.4	12.5	9.9	10.3	−0.8	−6.8	−23.4	3.8	−6.6
Poland	16.7	15.9	15.5	14.1	13.3	−5.1	−2.2	−9.8	−5.9	−5.6
Portugal	20.5	19.7	17.7	16.8	17.7	−3.8	−10.8	−5.7	5.5	−3.6
Republic of Moldova ^a^	96.9	85.8	83.9	80.6	72.5	−12.1	−2.3	−4.0	−10.6	−7.0
Romania	73.5	70.9	64.0	62.1	58.9	−3.6	−10.2	−3.0	−5.3	−5.4
Russian Federation ^a^	63.6	62.3	57.8	53.2	48.9	−2.1	−7.5	−8.2	−8.5	−6.4
San Marino			0.0	0.0	0.0			0.0	0.0	0.0
Serbia	10.9	9.8	8.4	8.3	7.2	−11.1	−14.6	−1.6	−13.8	−9.7
Slovakia	5.5	5.4	4.9	3.9	4.7	−2.8	−9.8	−22.9	20.1	−3.8
Slovenia	6.9	6.2	5.7			−9.8	−9.1			−9.0
Tajikistan ^a^	68.0	68.0	67.2	65.0	61.7	0.0	−1.1	−3.3	−5.3	−2.4
Turkey	16.9	15.9	15.2	14.5	13.9	−5.9	−4.6	−4.4	−4.3	−4.7
Ukraine ^a^	67.5	65.0	63.4	60.0	58.3	−3.8	−2.4	−5.6	−2.9	−3.6
United Kingdom	10.1	8.9	8.8	7.9	7.1	−12.9	−1.5	−10.4	−11.0	−8.6
**EECA**	**67.2**	**64.3**	**60.2**	**56.4**	**53.1**	**−4.4**	**−6.6**	**−6.6**	**−6.0**	**−5.7**
**Non-EECA**	**17.2**	**14.9**	**14.0**	**13.6**	**12.7**	**−14.6**	**−5.8**	**−3.4**	**−6.8**	**−7.4**
**All Countries**	**38.8**	**34.9**	**32.6**	**31.2**	**29.1**	**−10.6**	**−6.7**	**−4.4**	**−7.2**	**−7.0**
**b. Penitentiary Sector**										
**Country**	**Notification Rate of Incident TB Cases per 100,000 Population**					**Annual Change in Notification Rate of Incident TB Cases (%)**				
	**2014**	**2015**	**2016**	**2017**	**2018**	**2014–2015**	**2015–2016**	**2016–2017**	**2017–2018**	**Average**
Albania	76.9	0.0	49.2	147.7	56.8	−100.0		109.9	−95.5	−7.3
Andorra	0.0	0.0	0.0	0.0	0.0	0.0	0.0	0.0	0.0	0.0
Armenia ^a^	662.8	509.8	184.7	328.3	395.9	−26.2	−101.5	57.5	18.7	−12.1
Azerbaijan ^a^	1580.0	1115.8	1217.6	1266.5	1117.9	−34.8	8.7	3.9	−12.5	−8.3
Belarus ^a^		359.3	344.1	275.8	301.4		−4.4	−22.1	8.9	−5.7
Belgium	297.4	109.3	161.4	188.3	160.1	−100.1	39.0	15.4	−16.3	−14.3
Bosnia and Herzegovina	65.7	119.2			0.0	59.5				−100.0
Bulgaria	597.9	261.8	277.7	220.3	229.3	−82.6	5.9	−23.2	4.0	−21.3
Croatia	114.9		0.0							−100.0
Czech Republic	85.8	91.1	62.3	112.8	102.0	6.0	−38.0	59.4	−10.1	4.4
Estonia	181.8	296.6	281.6	107.1	200.0	49.0	−5.2	−96.6	62.4	2.4
France		91.3	97.8	95.2	96.7		6.9	−2.7	1.6	1.9
Georgia ^a^	973.8	844.0	610.7	625.0	473.4	−14.3	−32.4	2.3	−27.8	−16.5
Hungary	47.6		47.4		92.0					17.9
Ireland	37.3	0.0	0.0	0.0	0.0	−100.0		0.0	0.0	−100.0
Israel	24.1	9.9				−89.0				−89.0
Kazakhstan ^a^	1857.4	1164.9	967.1	1102.9	1002.3	−46.7	−18.6	13.1	−9.6	−14.3
Kyrgyzstan ^a^	2081.1	3038.5	2602.4	2238.7	1623.4	37.8	−15.5	−15.1	−32.1	−6.0
Latvia	1031.3	748.5	754.2	557.8		−32.1	0.8	−30.2		−18.5
Lithuania	660.0	557.4	513.6	818.3	718.8	−16.9	−8.2	46.6	−13.0	2.2
Luxembourg	152.4	0.0	0.0	0.0	0.0	−100.0		0.0	0.0	−100.0
Malta	0.0	0.0	0.0	0.0	0.0	0.0	0.0	0.0	0.0	0.0
Monaco	0.0	0.0				0.0				0.0
Montenegro	64.2	74.6				15.0				15.0
Netherlands	37.2	11.6	37.7	68.0	24.0	−116.3	117.5	59.0	−104.0	−10.4
Poland	192.4	218.2	302.3	230.3	230.8	12.6	32.6	−27.2	0.2	4.7
Portugal	378.5	421.9	261.3	237.7	348.9	10.9	−47.9	−9.5	38.4	−2.0
Republic of Moldova ^a^	1765.7	1809.4	2228.8	1275.4	1165.7	2.4	20.8	−55.8	−9.0	−9.9
Romania	699.0	567.8	579.0	466.0	553.3	−20.8	2.0	−21.7	17.2	−5.7
Russian Federation ^a^	1683.6	1543.3	1503.8	1335.9	1259.7	−8.7	−2.6	−11.8	−5.9	−7.0
San Marino			0.0	0.0	0.0			0.0	0.0	0.0
Serbia	145.8	145.8	103.1	75.0	37.0	0.0	−34.7	−31.8	−70.6	−29.0
Slovakia	284.3	209.7	211.9	219.2	204.0	−30.4	1.0	3.4	−7.2	−8.0
Slovenia	0.0	0.0	0.0			0.0	0.0			0.0
Tajikistan ^a^	1300.0	900.0	990.0	748.1	806.7	−36.8	9.5	−28.0	7.5	−11.2
North Macedonia	153.8	148.1	111.1	323.9	100.0	−3.8	−28.8	107.0	−117.5	−10.2
Turkey	89.4	76.9	75.3	53.8	55.3	−15.0	−2.2	−33.5	2.6	−11.3
Ukraine ^a^	1982.8	1875.7	1405.2	1222.2	1424.2	−5.6	−28.9	−14.0	15.3	−7.9
United Kingdom	43.2	35.1	29.0	23.8	28.3	−20.7	−19.1	−19.8	17.4	−10.0
**EECA**	**1703.9**	**1491.4**	**1403.6**	**1254.5**	**1192.8**	**−13.3**	**−6.1**	**−11.2**	**−5.0**	**−8.5**
**Non-EECA**	**158.2**	**128.1**	**132.5**	**116.3**	**107.4**	**−21.1**	**3.4**	**−13.1**	**−7.9**	**−9.2**
**All Countries**	**1084.6**	**920.7**	**861.3**	**759.9**	**682.2**	**−16.4**	**−6.7**	**−12.5**	**−10.8**	**−10.9**

EECA: east European central Asia; WHO: World Health Organization. Note: TB notification rate: number of new and relapse tuberculosis cases registered and reported per 100,000 population. ^a^ EECA country; in bold are the countries groups averages

**Table 3:** Notifications of new and relapse TB cases in the civilian and penitentiary sectors and relative risks (RR) of developing TB for inmates in relation to the civilian population, WHO European Region, 2014–2018.

Country	N&R Notified, *n*	N&R Notified, Civilian Sector, *n* ^a^	N&R Notified, Penitentiary Sector, *n*	RR (95% CI)	Reported Year
Albania	440	437	3	3.74 (1.20–11.64)	2018
Andorra	2	2	0	0	2018
Armenia ^b^	734	720	14	16.15 (9.53–27.38)	2018
Austria	619	619	0	0	2016
Azerbaijan ^b^	5038	4822	216	22.78 (19.89–26.09)	2018
Belarus ^b^	2359	2253	106	12.56 (10.34–15.26)	2018
Belgium	916	896	20	23.96 (15.39–37.30)	2017
Bosnia and Herzegovina	666	666	0	0	2018
Bulgaria	1323	1307	16	12.33 (7.54–20.18)	2018
Croatia	449	449	0	0	2016
Czechia	499	474	25	25.19 (16.85–37.66)	2017
Estonia	145	140	5	18.83 (7.72–45.90)	2018
France	4839	4774	65	12.93 (10.12–16.51)	2017
Georgia ^b^	2315	2272	43	7.86 (5.82–10.62)	2018
Hungary	737	728	9	6.33 (3.28–12.21)	2016
Iceland	8	8	0	0	2014
Ireland	294	294	0	0	2018
Israel	280	278	2	10.19 (2.50–41.45)	2015
Kazakhstan ^b^	1283	12,479	353	14.55 (13.10–16.17)	2018
Kyrgyzstan ^b^	6338	6198	140	13.24 (11.21–15.63)	2018
Latvia	543	522	21	20.68 (13.39–31.95)	2017
Lithuania	1268	1214	54	19.29 (14.70–25.30)	2017
Luxembourg	42	42	0	0	2018
Malta	42	42	0	0	2017
Monaco	0	0	0	0	2015
Montenegro	80	79	1	5.92 (0.82–42.50)	2015
Netherlands	791	784	7	5.22 (2.48–10.97)	2018
North Macedonia	217	214	3	9.71 (3.11–30.33)	2018
Poland	5196	5025	171	17.35 (14.90–20.20)	2018
Portugal	1856	1812	44	21.38 (15.86–28.83)	2018
Republic of Moldova ^b^	3022	2933	89	16.16 (13.1–19.93)	2018
Romania	15,586	11,472	114	9.35 (7.78–11.24)	2018
Russian Federation ^b^	78,258	70,967	7291	25.46 (24.85–26.07)	2018
San Marino	0	0	0	0	2018
Serbia	641	637	4	5.11 (1.91–13.65)	2018
Slovakia	228	210	18	56.66 (35.03–91.64)	2017
Slovenia	118	118	0	0	2016
Switzerland	531	527	4	9.16 (3.43–24.49)	2015
Tajikistan ^b^	5726	5605	121	12.98 (10.85–15.53)	2018
Turkey	11,576	11,421	155	3.97 (3.39–4.65)	2018
Ukraine ^b^	26,512	25,745	767	24.12 (22.46–25.90)	2018
United Kingdom	5248	5226	22	3.0 (1.98–4.57)	2017

CI: confidence interval; EECA: east European central Asia; N&R: new and relapse TB cases; RR: relative risks; TB: tuberculosis; WHO: World Health Organization. ^a^ Reference data for risk comparison. ^b^ EECA country.

**Table 4:** Favourable and unfavourable TB treatment outcomes for civilians and inmates on first-line drug treatment schemes, 2012–2016 cohorts, WHO European Region.

**a. Civilian Sector**							
**Country**	**Overall, *n***	**Favourable Outcome, *n* (%)**	**Unfavourable Outcome**			**Not Evaluated, *n* (%)**	**Last Reported Cohort**
			**Failure, *n* (%)**	**Died, *n* (%)**	**LTFU, *n* (%)**		
Albania	406	355 (87.4)	3 (0.7)	10 (2.5)	20 (4.9)	18 (4.4)	2016
Andorra	4	3 (75.0)	0 (0.0)	1 (25.0)	0 (0.0)	0 (0.0)	2016
Armenia ^a^	861	695 (80.7)	18 (2.1)	48 (5.6)	99 (11.5)	1 (0.1)	2016
Azerbaijan ^a^	1270	1048 (82.5)	71 (5.6)	16 (1.3)	113 (8.9)	22 (1.7)	2016
Belarus ^a^	2076	1849 (89.1)	44 (2.1)	111 (5.3)	68 (3.3)	4 (0.2)	2016
Belgium	955	782 (81.9)	0 (0.0)	58 (6.1)	63 (6.6)	52 (5.4)	2016
Bosnia and Herzegovina	907	505 (55.7)	13 (1.4)	64 (7.1)	3 (0.3)	322 (35.5)	2016
Bulgaria	1488	1270 (85.3)	15 (1.0)	122 (8.2)	81 (5.4)	0 (0.0)	2016
Cyprus	55	37 (67.3)	0 (0.0)	0 (0.0)	0 (0.0)	18 (32.7)	2016
Czechia	492	335 (68.1)	1 (0.2)	82 (16.7)	54 (11.0)	20 (4.1)	2016
Estonia	158	125 (79.1)	3 (1.9)	26 (16.5)	2 (1.3)	2 (1.3)	2016
Georgia ^a^	2666	2226 (83.5)	52 (2.0)	112 (4.2)	232 (8.7)	44 (1.7)	2016
Hungary	847	598 (70.6)	18 (2.1)	101 (11.9)	78 (9.2)	52 (6.1)	2015
Ireland	286	103 (36.0)	0 (0.0)	16 (5.6)	2 (0.7)	165 (57.7)	2016
Israel	261	216 (82.8)	1 (0.4)	19 (7.3)	8 (3.1)	17 (6.5)	2015
Kazakhstan ^a^	13,372	12,188 (91.1)	396 (3.0)	666 (5.0)	122 (0.9)	0 (0.0)	2015
Kyrgyzstan ^a^	5910	4838 (81.9)	108 (1.8)	351 (5.9)	591 (10.0)	22 (0.4)	2016
Latvia	560	477 (85.2)	0 (0.0)	52 (9.3)	28 (5.0)	3(0.5)	2016
Lithuania	1126	949 (84.3)	12 (1.1)	109 (9.7)	51 (4.5)	5 (0.4)	2016
Luxembourg	37	0 (0.0)	0 (0.0)	1 (2.7)	0 (0.0)	36 (97.3)	2014
Malta	49	37 (75.5)	0 (0.0)	1 (2.0)	3 (6.1)	8 (16.3)	2013
Monaco	0	0	0	0	0	0	2014
Montenegro	79	73 (92.4)	0 (0.0)	3 (3.8)	3 (3.8)	0 (0.0)	2015
Netherlands	850	741 (87.2)	0 (0.0)	30 (3.5)	33 (3.9)	46 (5.4)	2016
North Macedonia	260	230 (88.5)	1 (0.4)	18 (6.9)	10 (3.8)	1 (0.4)	2016
Poland	5904	3187 (54.0)	3 (0.1)	578 (9.8)	361(6.1)	1775 (30.1)	2016
Portugal	1833	1298 (70.8)	0 (0.0)	131 (7.1)	60 (3.3)	344 (18.8)	2016
Republic of Moldova ^a^	2909	2398 (82.4)	70 (2.4)	292 (10.0)	114 (3.9)	35 (1.2)	2016
Romania	12,304	10,578 (86.0)	193 (1.6)	996 (8.1)	518 (4.2)	19 (0.2)	2016
Russian Federation ^a^	64,591	47,524 (73.6)	3761 (5.8)	7098 (11.0)	3213 (5.0)	2995 (4.6)	2016
San Marino	0	0	0	0	0	0	2015
Serbia	722	583 (80.7)	6 (0.8)	64 (8.9)	26 (3.6)	43 (6.0)	2016
Slovakia	262	224 (85.5)	0 (0.0)	27 (10.3)	2 (0.8)	9 (3.4)	2016
Slovenia	129	105 (81.4)	0 (0.0)	21 (16.3)	0 (0.0)	3 (2.3)	2015
Tajikistan ^a^	5254	4690 (89.3)	103 (2.0)	223 (4.2)	195 (3.7)	43 (0.8)	2016
Turkey	11,851	10,323 (87.1)	31 (0.3)	698 (5.9)	311 (2.6)	488 (4.1)	2016
Ukraine ^a^	21,618	16,756 (77.5)	1326 (6.1)	2112 (9.8)	1339 (6.2)	85 (0.4)	2016
United Kingdom	5649	4554 (80.6)	0 (0.0)	315 (5.6)	262 (4.6)	518 (9.2)	2016
Uzbekistan ^a^	13,783	11,667 (84.6)	272 (2.0)	615 (4.5)	677 (4.9)	552 (4.0)	2012
**b. Penitentiary sector**							
**Country**	**Overall, *n***	**Favourable Outcome, *n* (%)**	**Unfavourable Outcome**			**Not Evaluated, *n* (%)**	**Last Reported Cohort**
			**Failure, *n* (%)**	**Died, *n* (%)**	**LTFU, *n* (%)**		
Albania	3	2 (66.7)	0 (0.0)	0 (0.0)	0 (0.0)	1 (33.3)	2016
Andorra	0	0	0	0	0	0	2016
Armenia ^a^	8	8 (100.0)	0 (0.0)	0 (0.0)	0 (0.0)	0 (0.0)	2016
Azerbaijan ^a^	194	177 (91.2)	2 (1.0)	10 (5.2)	5 (2.6)	0 (0.0)	2016
Belarus ^a^	47	45 (95.7)	1 (2.1)	1 (2.1)	0 (0.0)	0 (0.0)	2016
Belgium	18	11 (61.1)	0 (0.0)	0 (0.0)	4 (22.2)	3 (16.7)	2016
Bosnia and Herzegovina	0	0	0	0	0	0	2016
Bulgaria	22	21 (95.5)	0 (0.0)	0 (0.0)	1 (4.5)	0 (0.0)	2016
Cyprus	1	0 (0.0)	0 (0.0)	0 (0.0)	1 (100.0)	0 (0.0)	2016
Czechia	13	11 (84.6)	0 (0.0)	1 (7.7)	0 (0.0)	1 (7.7)	2016
Estonia	8	8 (100.0)	0 (0.0)	0 (0.0)	0 (0.0)	0 (0.0)	2016
Georgia ^a^	49	37 (75.5)	1 (2.0)	1 (2.0)	3 (6.1)	7 (14.3)	2016
Hungary	4	2 (50.0)	0 (0.0)	0 (0.0)	2 (50.0)	0 (0.0)	2015
Ireland	0	0	0	0	0	0	2016
Israel	2	2 (100.0)	0 (0.0)	0 (0.0)	0 (0.0)	0 (0.0)	2015
Kazakhstan ^a^	634	503 (79.3)	44 (6.9)	8 (1.3)	79 (12.5)	0 (0.0)	2015
Kyrgyzstan ^a^	162	130 (80.2)	4 (2.5)	9 (5.6)	18 (11.1)	1 (0.6)	2016
Latvia	32	28 (87.5)	1 (3.1)	1 (3.1)	1 (3.1)	1 (3.1)	2016
Lithuania	26	18 (69.2)	0 (0.0)	2 (7.7)	6 (23.1)	0 (0.0)	2016
Luxembourg	1	1 (100.0)	0 (0.0)	0 (0.0)	0 (0.0)	0 (0.0)	2014
Malta	0	0	0	0	0	0	2013
Monaco	0	0	0	0	0	0	2014
Montenegro	1	1 (100.0)	0 (0.0)	0 (0.0)	0 (0.0)	0 (0.0)	2015
Netherlands	14	6 (42.9)	0 (0.0)	0 (0.0)	4 (28.6)	4 (28.6)	2016
North Macedonia	3	2 (66.7)	0 (0.0)	0 (0.0)	1 (33.3)	0 (0.0)	2016
Poland	195	113 (57.9)	0 (0.0)	4 (2.1)	3 (1.5)	75 (38.5)	2016
Portugal	39	18 (46.2)	0 (0.0)	1 (2.6)	0 (0.0)	20 (51.3)	2016
Republic of Moldova ^a^	139	117 (84.2)	7 (5.0)	2 (1.4)	9 (6.5)	4 (2.9)	2016
Romania	155	145 (93.5)	0 (0.0)	6 (3.9)	4 (2.6)	0 (0.0)	2016
Russian Federation ^a^	8546	4811 (56.3)	841 (9.8)	325 (3.8)	328 (3.8)	2241 (26.2)	2016
San Marino	0	0	0	0	0	0	2015
Serbia	11	7 (63.6)	0 (0.0)	1 (9.1)	3 (27.3)	0 (0.0)	2016
Slovakia	17	17 (100.0)	0 (0.0)	0 (0.0)	0 (0.0)	0 (0.0)	2016
Slovenia	0	0	0	0	0	0	2015
Tajikistan ^a^	70	61 (87.1)	1 (1.4)	5 (7.1)	3 (4.3)	0 (0.0)	2016
Turkey	166	139 (83.7)	1 (0.6)	7 (4.2)	14 (8.4)	5 (3.0)	2016
Ukraine ^a^	997	478 (47.9)	434 (43.5)	28 (2.8)	48 (4.8)	9 (0.9)	2016
United Kingdom	26	16 (61.5)	0 (0.0)	1 (3.8)	2 (7.7)	7 (26.9)	2016
Uzbekistan ^a^	349	238 (68.2)	38 (10.9)	38 (10.9)	10 (2.9)	25 (7.2)	2012

LTFU: lost to follow-up; TB: tuberculosis; WHO: World Health Organization. ^a^ EECA country.

## Data Availability

Data available in a publicly accessible repository hosted by WHO https://www.who.int/teams/global-tuberculosis-programme/data. (accessed on 12 June 2021).
